# 

**Published:** 1871-05

**Authors:** 


### BOOK NOTICES.

"Fourth Series" of Henry Ward Beecher's Sermons, from verbatim reports, published by J. B. Ford & Co., 39 Park Row, 1871, being twenty-six discourses, printed exactly as delivered, on doctrinal and practical subjects in reference to the every-day affairs of life. No man of cultivation, no Christian can read these sermons without profit and instruction, compelling that searching self-examination which is always advantageous to the true religious life.

"The Christian Weekly," $2 a year, beautifully illustrated, 8 pages. Pub lished by the American Tract Society, 1408 Chestnut Street, Philadelphia. This excellent paper ought to find its way into every religious family as a means of attracting the attention especially of children, who so much delight in pictures, and who would be led thereby to read what is instructive and safe, because true and pure. The avalanche of demoralizing pictorials of the day loudly cries for antagonizing influences on the part of all Christian people of whatever creed.

RELIGIOUS NEWSPAPERS. — Taking up "The Presbyterian" of Philadelphia, the long time official organ of that great denomination, $2.50 a year, one is surprised to find such a large amount of valuable, instructive, and religious reading. In the issue for April 1st, there is a rich variety of reading matter, original and selected, short essays, foreign and domestic correspondence, besides commercial and political intelligence, with the news of the day from all parts of the country and the world, with industriously collected items of information on religious subjects, works and enterprises, not only in its own, but other Orthodox denominations. Considering the elevated class among which it circulates, the Philadelphia "Presbyterian" should be considered one of the very best vehicles for the advertisements of enterprising business men, who have to sell what is substantial, useful, and reliable.

"The Overland Monthly," $4 a year, 409 Washington Street, San Francisco, Cal., and 121 Nassau Street, New York, has within three years built up for itself a reputation by the ability of its original articles, of which it has good reason to be proud, and which commends it to the patronage of the educated families of our country.

"Scribner's Monthly," just six months old, has achieved a circulation of fifty thousand copies, distributed through the United States, Canada, Sandwich Islands, Australia, West Indies, South America, Europe, and reaching almost every spot where the English language is spoken, and all for the generally conceded reason that it well merits its extraordinary success. New York, $4 a year.

It is hard to tell how "The Phrenological Journal" at 389 Broadway, New York, manages to give so much reading matter, and so many interesting "illustrations," for only three dollars a year. But Mr. Samuel R. Wells, its editor, publisher, and owner, is such a persistent, indefatigable hard worker, and withal so much esteemed in New York, that the marvel can be accounted for. Temperance, Virtue, and Right Living are his mottoes, and for these he has battled right manfully and most unswervingly for these many years. And he has reward in a large circulation and in the appreciation of all good people.

"Hesperia," by CORA L. V. TAPPAN, published by the author, $1.75, and for sale at all the bookstores, is a book of poetry and blank verse, dedicated to the "Future Republic," where Liberty and Justice, Love and Fidelity, are to live and flourish. The first part is dedicated "To my Mother;" part second, "Fraternia," to Lucretia Mott; and other parts, respectively, to William Lloyd Garrison, Frederick Douglass, Wendell Phillips, Moketaveta, (the Sir Philip Sydney of the West), Walt Whitman (the Poet of the West). The Benediction is dedicated to Ulysses S. Grant, "the earnest patriot, the faithful servant of the people, the true friend of the oppressed and long abused Indian, the citizen soldier who prefers to exchange the laurels of war for the olive branch of peace." The book is got up in the very best style of "The Riverside Press."

"The Art of Printing," or Gutenberg, by Emily C. Pierson, published by Noyes, Holmes & Co., Boston, $2, is mechanically one of the most beautiful of books; it is a history of the art of printing put forth in a form more interesting than any novel, so full of remarkable facts, of striking incidents, and absorbing narrations, that the reader is scarcely able to lay it down until every page has been read. It is a memorial narrative of the man who brought the art of printing to light at Strasbourg and Mentz. Every intelligent printer, every notable author, every acknowledged scholar, every cultivated mind, owes to the industrious and talented authoress, a large measure of thanks for preparing such an interesting history of the art of arts. A hundred thousand copies ought to be sold within the season.

"Three Successful Girls," by Julia Crouch, gives more good reading in 382 pp. 12mo., from the teeming press of Hurd & Houghton, for a dollar and a half, than any volume issued in a long time. There is a practical interest in the whole narration which commends the book to all the families of the land. There is a tenderness in the dedication which goes directly to the reader's heart. "To my father and mother, whose affection for me, and interest in all my plans have never failed; to my brother and sister in this world and in the world beyond, and to the dear old home where we have all lived and loved and been happy, this book, with tender affection, is inscribed." There are twenty-seven chapters: the first "Washing Day," the last "At Home." Among the others are "The Orchard," "Good-by," "New York," "Plymouth Church," "Advertising," "Dark Clouds," "Sacrifice for Principle," "Fashionable Life," "After the Ball," etc. Reader, buy the book and read it.

The "American Tract Society" have issued "Judge Not," by John H. Raymond, LL. D., President of Vassar College, Poughkeepsie. "Hush," by Rev. Samuel Martin. Ten cents each. Also, "The New Life, or Counsel to Inquirers and Converts, by a Pastor," thirty-five cents, and "Sermons for the People," by that eminent and good man, Rev. Wm. S. Plummer, D. D., a dollar book, containing thirty-five discourses, which all Christian people can feed upon, and practicing, will grow in grace.

"The Feet," a useful and practical book, which contains valuable and practitical information for all who walk, and if heeded in time, would prevent an incalculable amount of suffering from corns, bunions, ingrowing toe-nails, etc. etc. A dollar book, published by Samuel R. Wells, 389 Broadway, New York.

"The Story of my Life," by Hans Christian Andersen, $2, by Hurd & Houghton, 13 Astor Place, New York. 569 pp. 12mo. It is a beautiful history of a beautiful and useful life, and well worthy a place in every cultivated family. Its moral teachings are good; its sentiments pure, practical, and Christian.

Our book, "Fun the Best Physic; or, Everybody's Life Preserver," $1.50, contains twelve hundred short paragraphs on health and disease of mind, body, and morals. Three thousand copies were sold before it was printed, and another edition of three thousand is in press. Sixteen thousand of "Health by Good Living" have been sold, but our publishers, Hurd and Houghton, 13 Astor Place, New York, will issue another edition promptly.

The Editor's office for medical consultation is at 40 Broadway, until 1 p. m., daily.

